# The Role of Long Non-Coding RNAs (lncRNAs) in the Development and Progression of Fibrosis Associated with Nonalcoholic Fatty Liver Disease (NAFLD)

**DOI:** 10.3390/ncrna4030018

**Published:** 2018-08-21

**Authors:** Amanda Hanson, Danielle Wilhelmsen, Johanna K. DiStefano

**Affiliations:** Diabetes and Fibrotic Disease Research Unit, Translational Genomics Research Institute, 445 N 5th Street, Phoenix, AZ 85004, USA; ahanson@tgen.org (A.H.); dwilhelmsen@tgen.org (D.W.)

**Keywords:** nonalcoholic fatty liver disease (NAFLD), nonalcoholic steatohepatitis (NASH), liver fibrosis, hepatic carcinoma, long non-coding RNA, epigenetics

## Abstract

Nonalcoholic fatty liver disease (NAFLD) encompasses a spectrum of conditions ranging from hepatic steatosis to inflammation (nonalcoholic steatohepatitis or NASH) with or without fibrosis, in the absence of significant alcohol consumption. The presence of fibrosis in NASH patients is associated with greater liver-related morbidity and mortality; however, the molecular mechanisms underlying the development of fibrosis and cirrhosis in NAFLD patients remain poorly understood. Long non-coding RNAs (lncRNAs) are emerging as key contributors to biological processes that are underpinning the initiation and progression of NAFLD fibrosis. This review summarizes the experimental findings that have been obtained to date in animal models of liver fibrosis and NAFLD patients with fibrosis. We also discuss the potential applicability of circulating lncRNAs to serve as biomarkers for the diagnosis and prognosis of NAFLD fibrosis. A better understanding of the role played by lncRNAs in NAFLD fibrosis is critical for the identification of novel therapeutic targets for drug development and improved, noninvasive methods for disease diagnosis.

## 1. Introduction

Long non-coding RNAs (lncRNAs) are emerging as important contributors to biological processes underlying the pathophysiology of human disease [1]. While the role of lncRNAs in cancer has been an area of active investigation for many years, the involvement of these molecules in the development of obesity, type 2 diabetes (T2D), and related comorbidities, such as nonalcoholic fatty liver disease (NAFLD), is relatively recent [2]. The purpose of this review is to summarize the experimental evidence demonstrating a functional role for lncRNAs in the development and progression of NAFLD fibrosis, focusing on work that was conducted in available animal models and NAFLD patients with fibrosis. We also discuss the potential applicability of circulating lncRNAs to serve as biomarkers for the diagnosis and prognosis of NAFLD fibrosis based on findings from liver fibrosis that were attributed to non-NAFLD etiologies.

## 2. Nonalcoholic Fatty Liver Disease: Prevalence, Clinical Management, and Risk Factors

Nonalcoholic fatty liver disease (NAFLD) encompasses a spectrum of conditions ranging from steatosis to inflammation, fibrosis, cirrhosis, and hepatocellular carcinoma (HCC) [3,4]. NAFLD is the major cause of chronic liver disease and it is associated with substantial morbidity and mortality in developed countries [5]. The global prevalence of NAFLD in adults is approximately 24%, with estimated rates ranging from 14% (Africa) to 32% (South America) [6]. Based on ultrasonography diagnosis, NAFLD is estimated to affect ~25% of the United States population aged 18 years and older [7], although other studies suggest that its prevalence may be even higher [8]. Estimates that are based on blood testing or International Classification of Diseases (ICD) 9/10 coding may also underestimate NAFLD prevalence [6]. Because routine NAFLD screening in primary care settings or obesity clinics is not recommended, many affected individuals likely remain undiagnosed [9], further masking the true prevalence of the disease. Despite differences in estimates of prevalence, however, the number of individuals with NAFLD is increasing worldwide, matching trends that are associated with obesity and T2D [6].

**Figure 1:**
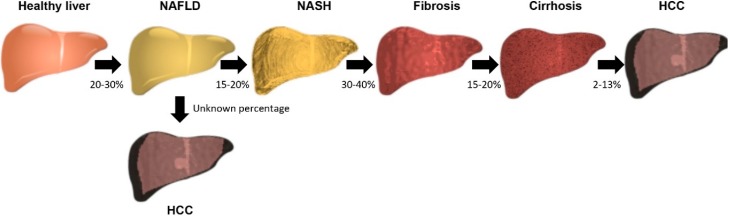
Stages of nonalcoholic fatty liver disease (NAFLD). Liver appearance in the various stages of the disease is schematized to represent the physical changes that accompany disease progression. The percentage of patients progressing from one stage to the subsequent stage is depicted below the arrows. NASH: nonalcoholic steatohepatitis, HCC: hepatocellular carcinoma.

NAFLD is a progressive disease that is characterized predominantly by excessive accumulation of triglycerides (i.e., steatosis) in the liver (Figure 1). Steatosis is defined as liver fat accumulation exceeding 5–10% by weight [10], but is usually estimated as the percentage of hepatocytes containing visible triglycerides that were observed by light microscopy [11]. While generally regarded as a benign condition, steatosis has been shown to progress to nonalcoholic steatohepatitis (NASH) and advanced fibrosis [12]. Approximately 15–20% of NAFLD patients develop NASH (steatosis with hepatocyte ballooning degeneration with or without fibrosis) [13] and 30–40% of NASH patients develop fibrosis [8]. In 15–20% of these patients, fibrosis can progress to advanced fibrosis or cirrhosis [14], and NASH patients with cirrhosis often develop HCC [15]. NASH is presently the second leading indication for liver transplantation in the United States [16], and it is predicted to be the most common indication within the next decade if the current trends of increasing NASH prevalence and decreasing hepatitis C virus (HCV) infection continue [17]. Liver transplantation is an effective treatment for NAFLD fibrosis; however, recurrent NAFLD is a common outcome within five years of transplantation [18], in part because the metabolic comorbidities that caused the disease in the first place typically remain unchanged.

The progression of NAFLD from steatosis to fibrosis has been modeled as a “multi-hit” process [19] with both environmental and genetic determinants [20]. While oxidative stress [21], pro-inflammatory cytokines [22,23], and immune response [24,25] have been associated with the coincident development of inflammation and fibrosis in NAFLD patients, the most common modifiable risk factors are obesity [26], dyslipidemia [27], and insulin resistance/T2D [28,29]. For example, steatohepatitis rates in normal weight, obese (body mass index (BMI) = 30.0–39.9 kg/m^2^), and extremely obese (BMI ≥ 40.0 kg/m^2^) NAFLD patients were 3%, 20% and 40%, respectively [30]. In T2D patients, NAFLD, NASH, and advanced NAFLD fibrosis rates have been estimated at 70%, 20%, and 5–7%, respectively [31]. NAFLD prevalence has been reported as higher in males [32] and to increase with age [33], although NAFLD in the pediatric population is escalating, which is due largely to the burgeoning incidence of obesity in children and adolescents [34]. In women, NAFLD was higher in patients with prior gestational diabetes compared to those without (14 vs. 6%, *p* < 0.01), suggesting that this condition is a risk factor for the development of fatty liver [35]. Infants born to obese women with gestational diabetes showed a 68% increase in intrahepatocellular fat at 1–3 weeks of age [36].

Evidence from familial aggregation [37,38,39], twin [40,41,42], and population-based [43,44,45] studies supports a heritable component to NASH, cryptogenic cirrhosis, and NAFLD. Interethnic differences in the prevalence of NAFLD and NAFLD fibrosis also indicate population-dependent susceptibility. In the United States, a number of studies have shown rates of NAFLD, NASH, and NAFLD-related fibrosis to be highest in Hispanics, followed by Asians, Non-Hispanic Whites, and African Americans [46,47,48,49], a distribution mirrored in the pediatric population [50]. A number of studies have identified genetic variants associated with the natural history and severity of NAFLD, NAFLD in lean individuals, risk stratification, and prediction of response to lifestyle or therapeutic intervention; the best characterized and replicated genes emanating from these studies include patatin-like phospholipase domain-containing protein 3 (*PNPLA3*) and transmembrane 6 superfamily 2 human gene (*TM6SF2*). Results from genetic studies in NAFLD [51] and NASH [52] have been reviewed in detail elsewhere.

The liver is comprised of four basic cell types: hepatocytes (parenchymal cells), hepatic stellate cells (HSCs), Kupffer cells (stellate macrophages), and sinusoidal endothelial cells. Hepatocytes comprise approximately 80% of the liver’s mass and perform the majority of hepatic functions, including those related to metabolism, synthesis, detoxification, and storage. Excessive free fatty acids are stored in hepatocytes, promoting the formation of toxic lipid metabolites, causing ballooning degeneration, and eventually leading to cell injury and death. Hepatocyte damage stimulates an inflammatory response, activating macrophages and HSCs [53]. Hepatocytes also contribute to the initiation and progression of fibrosis through complex processes involving nearby cells, which are triggered by hepatocyte injury [54].

HSCs are pericytes that are located in the perisinusoidal space of the liver, and they represent about 5–8% of the total liver cell content. Under normal physiological conditions, HSCs exist in a quiescent state, functioning as a storage site for retinols [55]. In response to hepatic injury, quiescent HSCs are transformed to a myofibroblast phenotype that is capable of secreting cytokines and other molecules to protect the liver. Loss of intracellular lipid droplets and the increased production of α-smooth muscle actin (ACTA2) and components of the extracellular matrix (ECM) accompany HSC activation. Following the resolution of injurious conditions, activated HSCs are removed through apoptosis and inactivation [56]. Under chronic conditions of hepatic inflammation, however, activated HSCs continue to produce ECM components, which eventually results in hepatic scarring. HSCs play a critical role in the development of liver fibrosis [57].

Despite a comprehensive understanding of cellular changes that occur during the development and progression of NAFLD fibrosis, the molecular mechanisms underlying these changes remain poorly understood. Given the association between fibrosis and increased liver-related mortality in NAFLD patients [58,59], it is important to identify and characterize specific mechanisms contributing to disease progression to develop new therapies for delaying, halting, or reversing hepatic fibrosis.

## 3. lncRNAs in the Development and Progression of NAFLD-Related Fibrosis

Little is known of the role of lncRNAs in the development or progression of NAFLD fibrosis, although growing evidence suggests that these molecules contribute to many of the pathophysiological mechanisms underlying the disease. In the following sections, we review the current literature from studies conducted in animal models and NAFLD fibrosis patients.

### 3.1. lncRNAs from In Vivo Studies of NAFLD Fibrosis Animal Models

Studies in animal models, mainly carbon tetrachloride (CCl_4_)-treated mice, an animal model of hepatic injury resembling NAFLD liver fibrosis, have largely focused on a candidate lncRNA approach followed by functional characterization in cells and pathway analysis. Summaries of the major findings from these studies follow.

#### 3.1.1. Maternally Expressed Gene 3 (*MEG3*)

Loss of the maternally expressed gene 3 (*MEG3*) lncRNA has been associated with an assortment of different human cancers [60]. One of the first investigations of lncRNAs in NAFLD fibrosis demonstrated that *MEG3* expression was decreased in livers from CCl_4_-treated mice, when compared to oil-fed control animals, and that *Meg3* expression diminished concordantly with progression of fibrosis [61]. These results were replicated in human patients with liver fibrosis of undisclosed etiology. Staining for *Meg3* in livers from fibrotic mice showed colocalization with *Acta2*, suggesting that HSCs represent the cellular source of this lncRNA [61]. Functional studies in the human HSC line, LX-2 cells, showed time-and dose-dependent downregulation of *MEG3* expression by transforming growth factor-β1 (TGFB1), while the overexpression of *MEG3* in LX-2 cells inhibited TGFB1-induced cell proliferation and promoted caspase-3-mediated apoptosis through mechanisms involving p53 and cytochrome c [61]. In contrast to these results, hepatic *MEG3* levels were significantly increased in liver fibrosis and NASH cirrhosis in human patients [62]. In our own unpublished work, we observed a slight upregulation in liver tissue from NAFLD fibrosis patients when compared to those with normal liver histology; however, we found significant downregulation of *MEG3* in activated LX-2 cells compared to quiescent cells, suggesting that this lncRNA may contribute to fibrosis through HSC-dependent mechanisms.

#### 3.1.2. Alu-Mediated p21 Transcriptional Regulator (*APTR*)

Using a targeted silencing RNA screening approach to discover human lncRNAs involved in cell proliferation, Negishi et al. [63] identified a novel lncRNA, *APTR*, which was shown to regulate cell cycle progression and cell proliferation. In an independent study, *Aptr* expression was found to be more than twofold higher in fibrotic livers of two animal models for liver fibrosis (CCl_4_ and bile duct ligation (BDL) mice) and in humans with liver fibrosis of undisclosed etiology [64]. Adenovirus-mediated *Aptr* knockdown in CCl_4_-treated mice reduced levels of Acta2 and collagen, type 1, alpha 1 (Col1a1), and attenuated liver fibrosis in treated animals. *APTR* knockdown in primary HSCs also decreased ACTA2 and COL1A1 mRNA and protein expression and attenuated TGFB1-induced upregulation of ACTA2. In individuals with cirrhotic liver of undisclosed etiology, serum *APTR* levels were approximately four-fold higher when compared to individuals with normal histology, and two-fold higher in patients with decompensated cirrhosis as compared to those with compensated cirrhosis, suggesting that serum *APTR* levels may also have value as biomarkers of severity for liver fibrosis. Because primary sequence conservation of lncRNAs is weak when compared to protein-coding genes [65], findings of similar *APTR* expression patterns in mice and humans, independent of the underlying etiology of liver fibrosis, is interesting and warrant further investigation. Additional studies, including characterization of the role of *APTR* in HSC activation, as well as analysis of serum *APTR* in a larger number of patients with a broader range of fibrosis (mild-severe), will be useful for determining the contribution of this lncRNA to fibrogenesis attributed to NAFLD.

#### 3.1.3. Metastasis-Associated Lung Adenocarcinoma Transcript 1 (*MALAT1*)

*MALAT1* contributes to cell proliferation, migration, and invasion in a number of human cancers, including HCC [66]. In CCl_4_-treated mice, hepatic *Malat1* expression was upregulated 5.4-fold and increased 5.9-fold and 2.7-fold in HSCs and hepatocytes isolated from CCl_4_-treated animals, respectively, when compared to control animals [67]. In primary HSCs that were isolated from C57BL/6 mice, knockdown of *Malat1* expression correlated with reduced levels of Acta2 and Col1a1, and reduced the appearance of the myofibroblast-like morphology characteristic of activated HSCs [67]. Further, knockdown of *Malat1* expression in CCl_4_-treated mice corresponded with a 54% decrease in collagen accumulation, suggesting that this lncRNA plays a role in the progression of liver fibrosis in mice. In that study, *Malat1* was shown to sequester miR-101b, leading to the activation of RAS-related C3 botulinum substrate 1 (Rac1) and promoting proliferation, cell cycle progression, and activation of HSCs. Levels of *MALAT1* and *RAC1* were increased 6.8-fold and 84%, respectively, in patients with liver cirrhosis of undisclosed etiology, suggesting that the same network may play a role in human fibrosis [67]. Hepatic *Malat1* expression was also reported to be increased in *ob/ob* mice, an animal model of T2D, as well as hepatocytes that were exposed to palmitate, implying complementary roles in other liver cell types [68]. As discussed more fully below, we observed elevated *MALAT1* expression in activated LX-2 cells, concordant with these findings, and demonstrated that *MALAT1* expression is modulated by hyperglycemia in HepG2 cells [69]. Together, these findings provide preliminary evidence supporting a role for functionally relevant differences in *MALAT1* expression in the development of liver fibrosis related to NAFLD.

#### 3.1.4. Plasmacytoma Variant Translocation 1 (*PVT1*)

*PVT1* expression is upregulated in a number of different cancers [70] and has been shown to mediate ECM accumulation in mesangial cells of the kidney [71]. Zheng et al. [72] reported 14.4-fold higher hepatic *Pvt1* levels in CCl_4_-treated mice compared with control animals and upregulation of three different *Pvt1* isoforms in HSCs at day 10 versus day 2 of culture. In that study, the authors showed that *Pvt1* knockdown in primary HSCs not only reduced cell proliferation, but also decreased transcript and protein levels of Acta2 and Col1a1 by 38–57%. Silencing of *Pvt1* in primary HSCs also corresponded with changes in levels of markers of epithelial-mesenchymal transition (EMT) process, namely E-cadherin, desmin, and vimentin, implicating a possible mechanism by which this lncRNA promotes liver fibrosis. In vivo studies demonstrated that *Pvt1* knockdown in CCl_4_-treated mice resulted in reduced hepatic collagen expression [72]. Characterization of a potential signaling network identified miR-152 as a driver of EMT and HSC activation through the inhibition of methylation of Patchd1 (PTCH1) and activation of the hedgehog pathway.

#### 3.1.5. Homeobox (HOX) Transcript Antisense RNA (*HOTAIR*)

Expression of *Hotair* was upregulated in livers from CCl_4_-treated mice when compared to oil-fed controls, and in activated HSCs, but expressed in low levels in HSCs from wild type mice, as well as mouse hepatocyte and macrophage cells lines [73]. *HOTAIR* levels were also elevated in fibrotic liver samples from hepatitis B virus (HBV) patients, and colocalized with *ACTA2*, suggesting that HSCs may be the primary source for *HOTAIR* in fibrotic liver. Functional characterization of the lncRNA showed that *HOTAIR* overexpression promoted cell proliferation and increased levels of *ACTA2* and *COL1A1*, as well as fibrosis-related genes, such as matrix metalloproteinase 2 (*MMP2*) and *MMP9* [73]. These results also indicated that *HOTAIR* may promote liver fibrosis through processes involving regulation of DNA methyltransferase 1 (*DNMT1*), *MEG3*, and the p53 pathway in HSCs, although further investigation of this lncRNA is needed to determine the role of *HOTAIR* in liver fibrogenesis.

#### 3.1.6. LncRNA-Cyclooxygenase 2 (*lncRNA-COX2*)

Cyclooxygenase 2 (*COX2*), which is also known as prostaglandin-endoperoxide synthase 2 (*PTGS2*), is the rate-limiting enzyme in prostaglandin biosynthesis and may be involved in liver cirrhosis [74]. A lncRNA located in proximity to *Cox2/Ptgs2*, *lncRNA-Cox2*, was investigated in CCl_4_-induced fibrotic mice based on evidence of its participation in the regulation of inflammatory genes [75]. Levels of both *Cox2* and *lncRNA-Cox2* were increased in CCl_4_-treated mice when compared to control animals, and a positive correlation was observed between the two transcripts and amount of tissue affected by fibrosis. Although these results were preliminary, and they have yet to be validated in liver samples from NAFLD fibrosis patients, they are the first to provide evidence supporting *lnc-COX2* involvement in hepatic fibrogenesis.

#### 3.1.7. Nuclear Enriched Abundant Transcript 1 (*NEAT1*)

Expression of *NEAT1* was upregulated in HCC, while its knockdown corresponded with decreased HCC cell proliferation, invasion, and migration though the regulation of heterogeneous nuclear ribonucleoprotein A2 [76]. *Neat1* expression was also observed to be elevated in whole livers and primary HSCs derived from CCl_4_-treated mice compared to oil-fed controls, while adenovirus-mediated knockdown of *Neat1* attenuated CCl_4_-induced liver fibrosis in these animals [77]. *Neat1* knockdown in primary HSCs resulted in a reduction of cell proliferation by 54% and decreased transcript and protein levels of Acta2 and Col1a1. In contrast, the overexpression of *Neat1* promoted HSC activation and increased Acta2 and Col1a1 levels, suggesting that this lncRNA plays a role in HSC activation. *Neat1* overexpression correlated with reduced levels of miR-122, which was found to mediate *Neat1* effects on HSC activation via a mechanism attributed to Kruppel-like factor 6 (*Klf6*). The *Neat1*-miR-122-*Klf6* axis was also found to function in hepatocytes and the levels of *NEAT1* and *KLF6* were increased, while miR-122 levels decreased in cirrhotic liver samples from patients with undisclosed etiology. We also observed elevated hepatic *NEAT1* levels in NAFLD patients with inflammation and advanced fibrosis [69], providing additional support for a role of this lncRNA in fibrogenesis in mice and humans.

#### 3.1.8. Genome-Wide Identification of lncRNAs in Animal Models of Liver Fibrosis

To our knowledge, two studies have applied a global approach to identify lncRNAs that were involved with liver fibrosis in animals. In the first study, Zhang et al. [78] used microarray analysis to profile lncRNAs in CCl_4_-treated mice and untreated control animals and identified 266 upregulated and 447 downregulated lncRNAs. Of these, the authors characterized a single lncRNA, which they named liver fibrosis-associated lncRNA1 (*lnc-LFAR1*), and found that it was most abundantly expressed in hepatocytes, followed by HSCs and Kupffer cells. *Lnc-FAR1* expression was downregulated in primary hepatocytes, but increased in primary HSCs of fibrotic mice. Knockdown of *lnc-FAR1* in primary HSCs corresponded with upregulation of 1195 mRNAs and downregulation of 1424 mRNAs, including many associated with ECM. Lentivirus-mediated knockdown of *lnc-FAR1* in CCl_4_-treated mice resulted in the attenuation of liver fibrosis and reduced levels of hepatic hydroxyproline content and serum levels of alanine transaminase (ALT) and aspartate transaminase (AST). In these animals, levels of pro-fibrogenic, pro-inflammation and pro-apoptosis genes were reduced. These findings were replicated in a BDL animal model of liver fibrosis, suggesting the effect of *lnc-FAR1* was independent of the type of liver fibrosis model. In mechanistic studies, the authors demonstrated that (1) *lnc-FAR1* promotes association of Smad2/3 with TGFβR1, which then phosphorylates Smad2/3 in the cytoplasm, and (2) *lnc-FAR1* binds directly to Smad2/3 to regulate transcription of a number of genes, including *Tgfβ*, *Pai*, *Acta2*, *Col1a1, Smad2*, *Smad3*, *Notch2*, and *Notch3*, leading to activation of the Tgfβ and Notch pathways. Together, *lnc-FAR1* was found to exert effects on HSC activation, hepatocyte apoptosis, and liver fibrogenesis in a mouse model of liver fibrosis. Because a clear human orthologue was not identified in this work, the critical next steps should involve establishing whether a version of the *lnc-FAR1* primary sequence, with a similar structure or function, exists in humans.

The second study performed RNA-sequencing in Sprague–Dawley rats treated with CCl_4_ and untreated animals and identified 231 out of 16,113 lncRNAs that were differentially expressed (fold-change > 2) between the two experimental groups [79]. The majority of the differentially expressed lncRNAs was not previously annotated, nor were the functions of these lncRNAs known. One lncRNA, NR_002155.1, which showed a 36.6-fold lower expression in fibrotic liver, was found to suppress HSC activation. While preliminary, these findings yielded a number of potential candidates for both follow-up functional characterization studies and the investigation of conserved counterparts in humans with hepatic fibrosis. Further, determination of serum NR_002155.1 levels and their correspondence with severity of fibrosis will be critical to assess the utility of this lncRNA to serve as a novel diagnostic biomarker for liver fibrosis.

Findings from animal studies conducted to date (Table 1) provide good preliminary evidence supporting dysregulated lncRNA expression and hepatic fibrosis. In some cases, possible mechanisms by which lncRNAs might contribute to fibrogenesis have been reported, but at this point, much more work is needed to understand the function of lncRNAs in the development of NAFLD fibrosis. Further, the majority of animal studies have thus far utilized CCl_4_, a hepatotoxin commonly used to induce hepatic fibrosis. Similar to hepatic fibrogenesis that was attributed to NAFLD in humans, CCl_4_ causes HSC activation, dysregulated ECM production and degradation, and progressive hepatic fibrosis [80]. However, CCl_4_-induced liver fibrosis results from the induction of hepatic lesions and the inherent toxicity of the compound alters liver homeostasis in a way that does not recapitulate NAFLD fibrogenesis in humans [81]. Despite this discrepancy, the replication of findings between CCl_4_-treated animals and patients with hepatic fibrosis (albeit of undisclosed etiologies) in a number of studies (Table 1) suggest that at least some lncRNAs identified in the CCl_4_ mouse model, namely, *Aptr*, *Malat1*, *Neat1*, and *Hotair*, may also be relevant to NAFLD fibrosis in humans.

### 3.2. Evidence from Studies of Patients with NAFLD Fibrosis

The number of studies of lncRNAs that have been conducted in humans with NAFLD fibrosis is relatively sparse when compared to those that were derived from animal models of liver fibrosis. In an early study, lncRNA expression was profiled using microarray analysis of liver samples from five patients with NAFLD and five patients without NAFLD; ~2000 differentially expressed lncRNAs were observed between the two groups [82]. While this study represented an important first step in delineating lncRNA expression patterns in NAFLD patients, it was limited by a very small sample size, a general lack of replication of findings of differential expression while using an orthogonal method of analysis for a subset of lncRNAs, and importantly, none of the NAFLD patients had liver fibrosis. A second study profiled 4383 lncRNAs in liver samples from 48 NASH patients, 11 patients with simple steatosis, and 23 healthy controls [83]. After adjusting for age, sex, and body mass index, expression of only one lncRNA, *RP11-484N16.1*, was significantly correlated with NASH grade, lobular inflammation, and NAFLD activity score, and nominally associated with fibrosis. The authors renamed this lncRNA *lnc18q22.2* based on its chromosomal position, 5 kilobases downstream from the cytokine signaling 6 gene (*SOCS6*). Analysis of *lnc18q22* expression in 30 different tissues and 67 different cell lines revealed the highest expression in liver tissue and the HepG2 human hepatoma cell line; expression was also detected in three other immortalized hepatocyte cell lines (Huh7, Hep3B, IHH) and primary human hepatocytes, but barely detectable or undetectable in HeLa cells and HEK293T embryonic kidney cells, respectively. *Lnc18q22.2* levels were also undetectable in human whole blood or plasma samples, excluding the lncRNA as a potential noninvasive biomarker of NASH. Knockdown of *lnc18q22.2* in HepG2, Hep3B, Huh7, or IHH cells yielded either a lethal phenotype or reduced cell viability, leading the authors to speculate that elevated *lnc18q22.2* expression functions as a protective mechanism against liver damage through the inhibition of hepatocyte death.

A third study performed RNA-sequencing of wedge liver biopsies to profile lncRNA expression in 24 NAFLD patients with normal liver histology, 53 NAFLD patients with lobular inflammation, and 63 NAFLD patients with severe fibrosis, identifying 4432 and 4057 differentially expressed transcripts in comparisons of normal tissue with lobular inflammation and fibrosis, respectively [69]. Comparison of profiles from lobular inflammation and advanced fibrosis revealed 3122 differentially expressed lncRNAs that were shared by the two histological groups, indicating that changes in lncRNA expression that corresponded with advanced fibrosis may begin with the onset of hepatic inflammation. Seventy-seven experimentally validated mRNA targets of differentially expressed lncRNAs that were common to inflammation and fibrosis were identified and analysis of these transcripts identified more than 100 pathways, including those involved in TGFB1 and TNF signaling, insulin resistance, and extracellular matrix maintenance. LncRNAs identified in animal models, discussed above, were also highly expressed in fibrosis relative to normal tissue in NAFLD patients, including *NEAT1*, *MALAT1*, and *PVT1*. Of these, *MALAT1* showed the strongest evidence for differential expression in fibrotic samples as compared to samples with either normal and inflammation histology, both of which showed similar levels of expression. *MALAT1* expression was also increased in activated LX-2 cells and upregulated by hyperglycemia. C-X-C motif chemokine ligand 5 (*CXCL5*) was identified as a potential target of *MALAT1*, and knockdown of *MALAT1* in liver cells corresponded with decreased expression of *CXCL5* transcript and protein. *CXCL5* expression was elevated in activated LX-2 cells, as is consistent with increased cytokine expression from HSCs in response to liver injury [84]. *CXCL5* has been previously shown to be upregulated in liver injury [85,86,87] and play a role in hepatocyte proliferation [85]. While additional studies will be necessary to delineate the relationship between *MALAT1* and *CXCL5* and determine the contribution from different cell types in the liver to the *MALAT1-CXCL5* pathway, these findings preliminarily suggest that functionally relevant differences in *MALAT1* expression may contribute to the development of NAFLD fibrosis through mechanisms involving inflammatory chemokines.

Concordant with these findings, a recent study reported that hepatic *MALAT1* expression was higher in NASH patients when compared to NAFLD patients with simple steatosis and controls [88]. Similarly, *MALAT1* abundance was greater in NAFLD patients with higher scores of ballooning degeneration, lobular inflammation, and presence of fibrosis, and was correlated with serum ALT and AST levels. Levels of *MALAT1* in paired liver biopsies taken at least five years apart showed an eight-fold increase in one patient who progressed from steatosis to NASH fibrosis and a 29-fold increase in another patient who progressed from fibrosis 0 (no fibrosis) to fibrosis 3 (advanced fibrosis). Given the presence of elevated *MALAT1* levels in HCC, this lncRNA may serve as a predictive marker of liver disease trajectory, from fibrosis to cirrhosis to HCC. A well-powered longitudinal approach with paired biopsies is needed to address this possibility.

### 3.3. Circulating lncRNAs in NAFLD Fibrosis

Histological examination of biopsied tissue is considered the reference standard for the diagnosis and staging of liver fibrosis; but patient discomfort, risk for complications, sampling error and bias, variability in histopathologic interpretation, and financial cost [89] have led to a growing interest in alternative noninvasive strategies [90]. Because lncRNAs are released into the circulation and show stability in blood, the use of these molecules as novel biomarkers of various human diseases, including HCC [91], has been increasingly explored [92,93]. For example, plasma levels of *RP11-160H22.5*, *XLOC_014172*, and *LOC149086* were correlated with tumorigenesis in HCC, while circulating levels of the latter two, *XLOC_014172* and *LOC149086*, were elevated even further in HCC patients with metastasis, suggesting that these two lncRNAs may serve as biomarkers for metastasis in HCC [94]. An independent study screened a training set of HCC-associated lncRNAs, including the three mentioned above, and reported that plasma levels of *LINC00152*, *RP11-160H22.5*, and *XLOC_014172* could effectively distinguish patients with HCC from chronic hepatitis patients and healthy controls [95]. A recent study reported that serum *MALAT1* levels could differentiate HCC patients (N = 30) from HCV-induced liver cirrhosis patients (*N* = 20) and healthy controls (*N* = 20) [96]. The median serum levels of *MALAT1* in the HCC group were higher when compared to the cirrhotic (2.54 vs. 0.70; *p* = 0.043) and healthy groups (2.54 vs. 0.32; *p* = 0.025); however, no significant differences were observed between the cirrhotic and healthy groups.

Liver fibrosis is sometimes considered a pre-cancerous precursor to the development of HCC, and it is possible that circulating lncRNAs predictive of HCC may also be altered in fibrosis. A recent study examined *lncRNA-ATB*, an lncRNA associated with liver fibrosis and vascular invasion in HCC [97], in HCV patients and found that plasma lncRNA levels were significantly correlated with liver fibrosis stage [98]. Serum levels of another lncRNA, *lincRNA-p21*, were negatively correlated with liver fibrosis stage in HBV patients [99]. In patients with alcoholic cirrhosis, plasma levels of *AK128652* and *AK054921* were inversely associated with survival, suggesting that these lncRNAs may serve as biomarkers to predict patient survival [100]. As discussed earlier, in patients with liver fibrosis of undisclosed etiology, serum *APTR* levels were increased in 15 patients with liver cirrhosis when compared to nine control individuals [64]. Despite promising findings of lncRNAs as biomarkers in HCC and non-NAFLD-related liver fibrosis, the potential of circulating lncRNAs to serve as diagnostic or prognostic biomarkers for NAFLD fibrosis remains an unexplored area of research. Investigation of lncRNAs from published reports in patients with liver fibrosis attributed to NAFLD/NASH, as well as unbiased approaches to identifying new lncRNAs, are urgently needed to evaluate the applicability of these biomarkers in this disease.

## 4. Conclusions

NAFLD fibrosis is an incompletely understood condition that is difficult to diagnose and for which there are no FDA-approved treatments. To date, investigations in animal models and NAFLD fibrosis patients remain preliminary, and they have largely focused on correlative associations between lncRNA levels and presence of fibrosis. Few studies have identified well-defined mechanisms by which specific lncRNAs promote fibrogenesis or utilized experimental strategies to directly address the function of lncRNAs in the fibrotic process. There is also a strong need for in-depth follow-up studies of lncRNAs identified in animal models in human cells and patient samples and improved in vivo models to investigate specific lncRNAs in the development and progression of NAFLD fibrosis, particularly as most of the studies have utilized the CCl_4_-induced fibrosis model, which does not exactly recapitulate the disease pathogenesis in humans. Other issues that complicate the study of lncRNAs in NAFLD fibrosis include the general lack of conservation across species, making in vivo studies more challenging, as well as the reality that lncRNAs appear to play a number of different functions, unlike miRNAs, which primarily serve to regulate gene expression through complementary base-pairing, making the path to functional characterization much more tortuous. Based upon these limitations, it is clear that a deeper understanding of the molecular mechanisms by which lncRNAs contribute to hepatic fibrogenesis, the cell types that are affected by dysregulated lncRNA function, the point in the disease process when fibrosis-related lncRNAs are becoming dysregulated, and the importance of the expression and molecular function behind these lncRNAs is urgently needed. Likewise, the investigation of circulating lncRNAs as biomarkers of NAFLD fibrosis is presently in its infancy, although because of the generally higher cell- or tissue-specificity of these molecules, their potential to predict disease progression may be promising. Much more research is needed to determine the applicability of lncRNAs to serve as accurate, noninvasive markers for early diagnosis of NAFLD fibrosis.

## Figures and Tables

**Table 1:** Summary of long non-coding RNAs (lncRNAs) identified in animal studies.

lncRNA	Expression	Replicated in Humans	Ref
***MEG3***	downregulated	Yes	[61,62]
***APTR***	upregulated	Yes	[63,64]
***MALAT1***	upregulated	Yes	[67,68]
***PVT1***	upregulated	No	[72]
***HOTAIR***	upregulated	Yes	[73]
***lncRNA-COX2***	upregulated	No	[75]
***NEAT1***	upregulated	Yes	[76,77]
***lnc-LFAR1***	upregulated in HSC	No	[78]
	downregulated in hepatocytes		
***NR_002155.1***	downregulated	No	[79]
